# Complications in Diabetes Mellitus: Social Determinants and Trends

**DOI:** 10.7759/cureus.24415

**Published:** 2022-04-23

**Authors:** Gaurav Venkat Cuddapah, Pujitha Vallivedu Chennakesavulu, Pradeep Pentapurthy, Mounika Vallakati, Akhila Kongara, Preethi Reddivari, Sindhu Singareddy, Kamala Pragna Chandupatla, Miryala Swamy

**Affiliations:** 1 Internal Medicine, Kamineni Academy of Medical Sciences and Research Centre, Hyderabad, IND; 2 Medicine, St. Martinus University Faculty of Medicine, Willemstad, CUW; 3 Internal Medicine, Prathima Institute of Medical Sciences, Karimnagar, IND; 4 Internal Medicine, Gandhi Medical College, Hyderabad, IND; 5 Internal Medicine, Malla Reddy Institute of Medical Sciences, Hyderabad, IND; 6 Medicine, MediCiti Institute of Medical Sciences, Hyderabad, IND

**Keywords:** economic stability, complications, income, social determinants, diabetes

## Abstract

Conditions that impact an individual's health are referred to as social determinants of health. Through a retrospective study (January 2017-February 2022) and statistical analysis, researchers looked at the relationship between social demands and type 2 diabetes mellitus (T2DM) diagnosis. All social demands, with the exception of childcare, were more typically documented in patients with T2DM. Prescription expense, conveyance, and health literacy were the domains with the greatest relationships. These results might help health systems and social service providers develop collaborations to help in certain areas.

## Introduction and background

Diabetes disproportionately affects racial and ethnic minorities, as well as people of little financial means in the United States, according to decades of study, with generally persistent patterns in these communities' higher incidence of diabetes complications, diabetes risk, and mortality. Social determinants of health (SDOH) are becoming more important as health care shifts to stress on inhabitants' health consequences and value-based care has emerged as a critical intervention target for achieving health equality. The latest coronavirus epidemic has brought to light the disproportionate vulnerabilities that racial and ethnic minorities, as well as underprivileged communities, face. Professional groups have issued comments on SDOH in the aftermath of pandemic and racial inequity incidents in the United States, with activities focusing on improving these determinants at the policy level, individual level, and organizational level. Because of the disease's prevalence, economic implications, and disproportionate population burden, understanding and minimizing the effect of SDOH is a top priority in diabetes research [1].

The American Diabetes Association (ADA) has issued a technical proclamation on the socio-ecological aspects that influence pre-diabetes and type 2 diabetes mellitus (T2DM). Pre-pandemic, the ADA summoned the existing diabetes writing committee to research connections of SDOH to diabetes risk and consequences, as well as the impact of the project target specific SDOH augmentation on diabetes consequences, with the intent of deeper knowledge and progressing chances for diabetic individuals improving health via SDOH. The first section of this article shows an insight into essential concepts and SDOH approaches. The emphasis of the review is on investigations of individuals with diabetes and their SDOH, i.e., socioeconomic status, the physical and social surroundings of one's community, the food environment, health care, and the social setting. The evaluation closes with suggestions for cross-sector collaboration in health care and the community [2].

Methods

Data Source and Strategy

Article search was performed in PubMed, Google Scholar, and Scopus. The following Medical Subject Headings (MeSH) and keywords have been applied: "diabetes" OR "diabetes mellitus" OR "social determinants of health" OR "SDOH" OR "diabetes socioeconomic status" OR "income" OR "education" OR "occupation" OR "housing" OR "toxic environmental exposures" OR "environmental risk factors" OR "environmental exposure" OR "social context" OR "global trends" OR "diabetes complications" OR "diabetes outcomes" OR "macrovascular complications" OR "microvascular complications" OR "mortality" OR "non-cardiovascular mortality" OR "retinopathy" OR "ESRD" OR "health care interventions" OR "emerging complications."

Study Selection and Eligibility Criteria

Authors conducted extensive screening after identifying relevant papers. After screening titles and abstracts, full-text articles were evaluated for eligibility. On relevant full-text articles, a quality evaluation check was performed. Only publications that passed a 70% quality rating check were included in the study. We looked for studies on diabetes mellitus socioeconomic factors and trends in complications that were published in the English language between 2017 and 2022. The existence of a substantial body of research to document the effect of the determinant on diabetes and representation within one or more current SDOH frameworks was used to determine inclusion in this study. Nomenclature in the relevant study uses T2DM, type 1 diabetes mellitus (T1DM), and "diabetes" (either a mixed T2DM and T1DM sample or diabetes case ascertainment procedures that do not allow determining clinical diagnostic type) to refer to the various forms of diabetes.

## Review

Diabetes SDOH

SDOH comprise factors such as housing, food security, social security, and economic stability, as shown in Table 1.

**Table 1:** Social determinants of health (SDOH)

Economic stability	Neighborhood	Food	Health	Social factors
Education	Housing	Food security	Quality	Support
Income	Built environment	Food access	Affordability	Insurance
Occupation	Toxic environmental exposures	Food availability	Access	Social capital
Policy		Insecurity in the food supply		

Diabetes Social and Economic Circumstances

It is a multidimensional term that takes into account educational, economic, and occupational considerations. At all levels of socioeconomic status (SES), it is an invariably significant prophet of disease outset and advancement for numerous diseases, including diabetes. SES is linked to almost every well-known SDOH. It has to do with people and communities' access to physical resources such as housing, health care, nutritious food, and transportation, as well as a social phenomenon like social engagement, political power, control, and awareness. Even though the three aspects of SES are interconnected, each one has its respective set of health effects. Every feature can be evaluated individually or in a group. Calculating a person's income, for example, is a typical technique to assess their financial situation. It is, however, influenced by the individual's household income as well as the community's income level so as representation for a person's monthly wage [3].

Income level also serves as a unique property at the census level, indicating the demographics and funds allocated in a given location. The number of years spent in school or the highest degree earned may both be used to assess educational achievement. Individually, at home, and in the community, it may be quantified. Disparities in educational quality, which may be relevant in determining SES, are not taken into account by the number of years spent in school. Amongst African Americans and low-income Whites, literacy has evolved as a metric of quality of education, and it may be more pensive of SES than years of schooling. A component of Healthy People 2020 is health literacy, which is linked to literacy and is context-specific. Literacy is also incorporated as part of SDOH. Occupation is a complicated subject in and of itself. Work status, stability, job kind, and working circumstances have all been considered. Employees of significant corporations have their work situations, qualifications, and salary recorded in occupational tiers of job categories [4].

Diabetes Incidence, Prevalence, and Outcomes as a Function of Socioeconomic Status

Up to the canopy of the SES hierarchy, aspects such as family revenue, education, job, and policy all recreate a role in the preponderance and consequences of diabetes. Those from economically disadvantaged backgrounds are much more likely to develop T2DM, suffer more comorbidities, and die earlier than those with higher SES. A person's wealth, educational achievement, and work status all play a part in their risk of acquiring T2DM. People with lower levels of wealth and literacy have been the primary focus of the research because of the steeper gradient toward the bottom of the distribution.

Income

The prevalence of diabetes rises linearly from the wealthiest to the poorest people. Based on information from the National Health Interview Survey (NHIS), researchers found that diabetes was more common among those with lower incomes (as measured by the income-to-poverty ratio). In terms of diabetes prevalence, people in the middle-class, near-poor, and poverty brackets had a 40%, 74.1%, and 100.4% relative percentage difference as compared to those in the high-income brackets. As a result of these findings, income gaps in diabetes prevalence are becoming more pronounced than they were before this period. SES plays a major role in determining local disparities in diabetes prevalence. People with T2DM have considerably higher rates in census tracts with substantially lower household earnings, fewer high school graduates, more single parents, and overcrowding. The probability of developing T2DM in pre-diabetic grown-ups was increased in census tracts where residents had lower levels of schooling, lower yearly earnings, and a more elevated proportion of households receiving Supplemental Nutrition Assistance Program (SNAP) benefits [5].

Those who live in impoverished neighborhoods have a twice more heightened risk of developing diabetes than those who live in non-poor neighborhoods, and those who live in non-poor neighborhoods have a redoubled risk of developing diabetes. A race-to-poverty-place gradient was also found. Poor Whites in low-income areas had the highest diabetes risk, followed by inferior Blacks in low-income areas and non-poor Blacks in low-income areas, and then poor Whites in non-low-income areas. There was a two-fold greater risk of diabetes-related death among those with T2DM who had a household revenue below the national poverty threshold. In addition, diabetes puts those with T1DM at a higher chance of death. Among those with T2DM, a meta-analysis found a negative relationship between income and hemoglobin A1c (HbA1c) levels, with a difference of 0.20% between those with higher and lower HbA1c. Poor revenue is associated with a more elevated incidence of diabetic ketoacidosis (DKA) and a higher HbA1c in both adolescents and young adults with T1DM [6]. This is especially true for children of color and those from poorer socioeconomic backgrounds.

Education

Adults' age-adjusted diabetes diagnosis rates rise in direct proportion to their level of education. There were 7.8 cases of diabetes per thousand grown-ups with only a high academy diploma, compared to 5.3 cases per thousand adults with a university degree or higher. Adult diabetes prevalence in the United States is likewise adversely correlated with educational attainment in a stepwise approach. Diabetic patients in the United States were diagnosed at a higher rate among individuals who had not completed high school than among those who did. Diabetic complications are less likely in people with higher levels of education [7].

Temporal crazes in diabetes preponderance at different educational levels demonstrate a growing disparity in prevalence with educational attainment. Gradually increasing levels of education are associated with an increased risk of diabetes-related death. Diabetes mortality is more than twice as high in those with only a high school diploma as it is in those with a college degree or higher. Patients with T1DM who did not attend college had a death rate three times higher than those who did. A meta-analysis found a 0.26% difference in HbA1c between people with high and low scholastic levels. Those with lower educational levels had greater HbA1c. A meta-analysis of 61 studies involving 18,905 people with T1DM or T2DM indicated that health literacy was related to lower HbA1c levels and more understanding of diabetes, but not with more frequent self-management behaviors [8].

Occupation

Even though a majority of this analysis was conducted farther from the United States, meta-analyses and systematic reviews have looked at a variety of career characteristics of diabetes risk. A meta-analysis of the linkages between employment insecurity and diabetes showed a connection between increased job insecurity and higher diabetes threat. According to a meta-analysis, unemployment is connected to an increased risk of prediabetes and T2DM. Shift work has been related to a higher risk of diabetes than normal daytime employment. Longer work hours were linked to a higher prevalence of diabetes in those with lower socioeconomic levels, but not in those with higher socioeconomic positions, according to a meta-analysis. According to population-based research in the United States, transportation workers had the highest risk of diabetes, while physicians had the lowest rate [9].

Policy

Diabetic insurance coverage and access to health care have been examined as a result of the Affordable Care Act. From 2009 to 2016, 770,000 more people between the ages of 18 and 64 years with diabetes received health insurance, with large gains among Whites, Blacks, and Hispanics, those making less than $35,000 per year, and people of all educational levels. Healthcare costs for diabetic adults in the poorest income category fell from 6.3% to 4.8% of their income. The Affordable Care Act resulted in greater healthcare coverage, improved diabetes management, and enhancing health quality for more persons in expanded Medicaid states compared to non-Medicaid expansion states. In addition, more people in Medicaid expansion states had higher rates of diabetes detection and diagnosis [10].

Interventions by SES

Despite the common occurrence of income and salary fluctuations, as well as job moves and losses, there is no evidence that these factors influence diabetes outcomes. Similarly, no diabetes outcomes have been recorded from programs aimed at underprivileged children and their families' living wages, early childhood schooling, scholastic quality, or access to education. Low-literacy adaptations and health literacy and numeracy tools are successful in enhancing diabetes awareness and self-care among racial/ethnic minority individuals with T2DM, according to studies evaluating diabetic self-management interventions. It was found that when compared to standard clinical treatment, literacy-sensitive treatments were linked to a small but statistically significant drop in HbA1c in patients independent of health literacy level. An evidence-based approach to behavioral self-management for racial/ethnic minorities with T2DM and inferior literacy and health literacy may be necessary to obtain significant outcomes. Because SES has long been identified as a key threat factor for diabetes and its consequences, the literature does not adequately address the role that changes in SES play in diabetes risk and outcomes [11].

Diabetes and the physical environment

It is becoming increasingly popular to study health outcomes in the context of one's living environment. It has been suggested that the historical and contemporaneous patterns of residential segregation based on socioeconomic position, race, and ethnicity are a product of the political and socioeconomic backdrop that established imprints of unequal resource allocation. It is proposed that comprehending the confluence of these environmental elements is critical to avoiding diabetes inequalities. Housing, the built environment, and environmental exposures all affect the neighborhood and physical environment [12].

Housing

There are many indicators of economic stability that are linked to housing stability. Having difficulty paying rent, being evicted or relocating frequently, spending more than 50% of one's earnings on rent, and residing in overcrowded conditions (traditionally portrayed as harboring more than one person per room) are all indicators of housing instability. The most extreme type of housing instability is homelessness. Homelessness is described as "the absence of a regular nighttime dwelling or the presence of a primary nocturnal residence that is a transient shelter or other space unsuitable for sleeping." As of 2020, the United States government identified 567,715 homeless people, or 17 out of every 10,000 people in the country; African Americans made up 40% of those suffering homelessness, while Hispanics and Latinos accounted for 22% of those encountering homelessness in the United States in 2020, according to a report from the Department of Housing and Urban Development [13]. Preventive treatments and self-care are difficult because of the instability, resulting in poorer chronic health condition control, increased use of emergency room visits, and a greater risk of complications, according to conceptual models relating housing insecurity to poor health.

Diabetes and Housing Instability

Uncertainty surrounds the prevalence of diabetes and whether or not it differs from that of non-homeless people in the United States. The lack of a widely accepted definition and an assessment tool for home insecurity is a major shortcoming in the profession. Housing instability has been correlated to a higher commonness of diabetes in lower socioeconomic groups; however, it is not clear if this is a direct link between housing instability and diabetes. Although both homeless and non-homeless people have an estimated 8% prevalence of diabetes, a comprehensive study found no evidence that the risk among the homeless was higher than the general population. More than one-third of people with diabetes are homeless, according to new research based on data from treated patients at community health clinics around the country. Self-reporting of a diabetic emergency care admission or hospitalization was more frequent among those with T2DM and housing crises, the findings show [14].

In a healthcare system, a cross-sectional study found a link between higher rates of outpatient diabetes treatment and people with diabetes experiencing housing instability. However, a separate study has connected poor health consequences and limited access to medical care to housing instability but not to diabetes. The Department of Veterans Affairs (VA) healthcare system conducted a long-term study and found that homelessness was linked to greater adjusted probabilities of having an HbA1c of 8.0% and 9.0%. Low-income people's inability to access diabetes medication has been linked to their living in unstable housing, according to studies. As food insecurity increased, so did diabetes self-efficacy, which dropped proportionally as housing instability rose. Distressed housing has been found to hinder self-care, as well as the acquisition of insulin and other diabetes supplies and equipment, as well as the consumption of food that is nutritionally balanced. All of these factors must be taken into account when deciding on a medication, including the ability to properly store medications and diabetes supplies and the expense of prescriptions. Homeless people's worries about diabetes treatment are summarized in this study [15].

Diabetes Outcomes and Housing Instability

Affordable housing is among the most difficult societal needs to meet when it comes to health. There are some high-quality data on housing therapies, even though there are few housing intervention studies that report diabetes results. Housing and Urban Development teamed with behavioral scientists and additional national agencies to conduct the Moving to Opportunity Demonstration Project (MTO) to examine the effects of moving from a high-poverty area to a low-poverty area on various outcomes. A total of 4,498 women with children in government-aided housing in high-poverty census areas were randomly assigned to one of three study arms between 1994 and 1998 by MTO. Of women who participated in the study, 1,788 were issued Section 8 vouchers that could only be used in low-poverty regions (census tracts where fewer than 10% of the population is poor) as well as counseling and support in finding a private rental property. Additionally, each of the 1,312 women received a typical briefing from their local program. Although they did not receive vouchers, MTO project-based help was provided to the 1,398 women in the control group as a backup plan. Those who received vouchers have the option of using them or not [16].

Compared to the control group, those who moved to low-poverty census tracts saw a 21.6% relative decline in elevated HbA1c prevalence, with a 4.31 percentage point absolute difference, according to follow-up studies conducted from 2008 to 2010. Another 13.0% drop in the prevalence of BMI of $35 kg/m2 was observed in the low-poverty group, as well as a 19.1% drop in the prevalence of BMI of $40 kg/m2. A similar set of coupons and controls had been utilized previously. Children and adults in low-poverty census tracts benefited from MTO's greater focus on improving the quality of housing, education, job, and income. Researchers found that persons who were given housing vouchers were less likely to be diabetic, have lower paces of intense obesity, and improved mental fitness outcomes over a decade to 15 years after receiving the vouchers. A randomized meta-analysis of tests that delivered low-barrier accommodation help to homeless people found significant reductions in healthcare utilization. If living conditions can be improved, diabetes treatment may be more widely available [17].

The Collaborative Initiative to Help End Chronic Homelessness could provide stable homes, primary care, and mental health care for chronically homeless adults. A higher proportion of unplaced individuals accessed assessment and management services than placed individuals did. When compared to those who were not already diabetic, people in the intervention group had higher HbA1c and lipid levels, but new cases of diabetes were less common. Having a place to call home has been associated with better control of diabetes in studies because it allows for better prioritization and routinization of treatment. This shows how the advantages of a supporting and stable way of life can be used to treat and prevent diabetes. A realistic qualitative analysis of the influence of transitioning to rental-assisted lodging on low-income, housing-insecure adults with T2DM found that rental assistance gave people more environmental and monetary power over their lives, allowing them to stick to their diabetes routines and allocate financial aid to diabetes care [18].

Exposure to Toxic Components

Toxic environmental exposures may occur naturally or as a result of human activity. Air pollution, environmental toxicants, and ambient noise, all of which have been associated with diabetes, are disproportionately exposed among disadvantaged communities in the United States, with groupings that produce the least pollution having the highest exposures. Residential segregation and differences in commodities and services are elements that contribute to disparities in hazardous environmental exposures and opportunities. Poor regulatory enforcement, a lack of response to community concerns, and the closeness of disadvantaged neighborhoods to nearby pollution sources are all possible factors. Arsenic and other metals/metalloids, pesticides, and dangerous chemicals are found in millions of people's drinking water from unregulated remote wells in rural and suburban regions, including Native American Indian villages. Individuals may be exposed to endocrine-disrupting chemicals via food packaging and fast-food dining, both of which are widespread in low-income communities. Chemicals released during microwave heating from plastic packaging, higher urine phthalate levels connected to fast food, and elevated bisphenol A levels in urine linked to canned foodstuffs are just a few instances. Underprivileged people are disproportionately exposed to phthalates and metal-containing personal care and cosmetic products [19].

Environmental Diabetes Risk Factors

National Toxicology Program assembled a multinational workshop in 2011 to assess practical and epidemiological research on the link between environmental pollutants and obesity, diabetes, and metabolic syndrome. For arsenic exposure, the relative risk of developing T2DM ranged from 1.11 to 10.05 in multiple studies. A growing number of chemical groups or individual chemical groups are being studied in systematic literature reviews and meta-analyses. Persistent organic pollutants like arsenic, phthalates, and bisphenol may increase the risk of developing diabetes in persons who are exposed to them. According to a few epidemiological studies, exposure to air pollution appears to increase the sensitivity of animals to insulin resistance and T2DM. “People who live in areas with higher levels of air pollution may be at greater risk of developing diabetes” [20], was a conclusion according to these studies.

Studies have shown that environmental exposures can raise the risk of cardiovascular disease (CVD) in patients with diabetes. Many studies have linked air pollution to health problems. Particulate matter concentrations of 10 mg/m3 and aerodynamic width of 10 mm were linked to a 2.01% upsurge in hospitalizations due to CVD in Medicare patients with diabetes, compared to 0.94% in those without diabetes. Compared to non-diabetic persons, diabetes patients have a greater risk of stroke death when short-term increases in pollution exposure occur. Diesel exhaust particles worsened the cardiovascular vulnerability of diabetes-affected rats in one investigation. In natural investigations in human populations, air pollution increased vascular inflammation and reactivity in diabetic patients in comparison to individuals who did not have diabetes. The presence of metals can also be seen. Diabetes was found to increase the threat of CVD in the Strong Heart Study, which included American Indians and tracked participants from 1989 to 1991. Disodium edetate chelation improved cardiovascular outcomes in diabetic patients in a clinical study [21].

Diabetes and Exposure to the Environment

Infrequent investigations have examined the impact of population-based or clinical therapy on diabetes prevention or management when it comes to environmental exposures. Diabetics living in polluted environments face an increased risk of complications from their disease, and this could open up new possibilities for treatment and prevention, especially for the most vulnerable among us. Four studies executed in four distant nations before and after the installation of smoke-free regulations showed a decrease in the number of pregnancies, but the long-term benefits have yet to be proved. The most successful therapeutic interventions will be at the population level, via guidelines and principles, with an emphasis on neglected groups because people have minimal authority over environmental factors. CVD has been linked to lower levels of air pollution and metal exposure; however, the benefits for diabetes have yet to be proved. A greater amount of clinical research is required to evaluate intermediate methods such as exposure screening and recommendations to test air or water as well as minimize known exposure sources and implement home therapies [22].

Diabetes and the Food Environment

In addition to the bodily existence of food that influences an individual's diet, the placement of food marts, food assistance, and any other physical commodity through which food can be procured, the food environment encompasses any interconnected system that permits access to foodstuffs. There are a variety of factors that influence a person's food and beverage choices as well their nutritional state. Known as the consumer-level environment or the community food environment, it influences food choices and diet quality through interactions between the two. Accessibility, availability, affordability, and quality are all part of the food environment. Food environment quality is particularly crucial in marginalized communities with limited access to grocery stores and nutritious foods but many fast food outlets and energy-dense items, as well as physical risks. Policies such as redlining and racial segregation in the past may have had an impact on the food environment [23].

Diabetes outcomes and the food environment

Accessibility to Food

It has been shown that the prevalence of T2DM and factors such as food accessibility, availability, and geography are linked in a cross-sectional study. In total, 3,128 counties across the country were surveyed to determine how easy it is to get food. Greater attainability of full-service meals and grocery marts was associated with a decrease in the prevalence of T2DM in both urban and non-metro areas, whereas a decrease in the attainability of fast food and convenience marts was associated with an increase in the prevalence of T2DM. Another study found that persons with diabetes were less likely to frequent full-service restaurants, whereas those who frequent fast-food joints were more likely to have the disease. Because of its county composition, this study found differences in the associations between a variety of food environment characteristics such as SES, demographics, food availability, and diabetes preponderance, which highlights the difficulty of interpreting patterns among variables such as SES, demographics, and also food availability. Diabetes preponderance and incidence have been linked in several observational, long-term studies to community resources in general and food environment access and attainability in particular [24].

Fast food outlets and convenience stores have been associated with an increased risk of T2DM, although the perceived healthfulness in the eating environment has been linked to a decreased risk of the disease. The density of grocery stores, on the other hand, did not appear to be associated with an increased risk of T2DM. Meta-analyses were not possible because of the wide range of topics covered in the publications. When researchers analyzed the Jackson Heart Study data on 3,700 African-Americans, they found that a larger density of adverse food outlets was related to a 34% increase in T2DM incidence after correcting for individual-level risk factors [25]. While living in zip codes with a larger percentage of poverty and higher walkability ratings was connected to an increased risk of diabetes, living in zip codes with a higher supermarket density was linked to a lower risk of T2DM in an employee cohort. Although the findings varied according to the methodology used, research has established a correlation between a lower prevalence of T2DM and long-term exposure to domestic environments that promote nutritious food and physical activity (PA). Diabetes risk is also linked to a person's diet and activity habits, according to previous research [25].

To examine population density-specific clusters of neighborhood indicators, researchers integrated measurements of neighborhood food and PA settings with weight-related outcomes from the Coronary Artery Risk Development in Young Adults (CARDIA) study. Diet quality was better in low-population density areas where there is greater variance in food and PA resources. In more densely populated places, a group with comparatively more realistic food/specialty shops, fewer convenience stores, and more PA resources was linked to better diet quality. BMI and insulin resistance were not connected to neighborhood clusters, nor were fast food consumption, walking, bicycling, or running. From 2007 to 2013, researchers used data from the New York City A1C Registry to examine the effect of home socioeconomic, nutritional, and physical settings on diabetic glycemic management. Healthy food options and walkability were associated with improved glycemic control and less time spent by residents of more affluent communities [26].

Food Affordability

According to a study, people with lower SES are more likely to be influenced by significant price differences between healthy and unhealthy items. In a long-term study, they looked at food affordability and neighborhood food costs, as well as the link between T2DM and insulin resistance, to determine which foods were more affordable. Although it was found that higher prices for excellent foods compared to poor foods decreased consumption of a high-quality diet, no link was found between diabetes incidence or prevalence and these higher costs. Further research is required in this area [27].

Insecurity in the Food Supply

Food insecurity is defined as a family's inability to maintain an active and healthy lifestyle at all times due to a lack of adequate food supply. Around 20% of diabetic patients report home food insecurity as a threat factor for deficient diabetes care. Food insecurity has been linked to a worsening of T2DM outcomes, according to experts. Food insecurity is linked to a higher HbA1c level, according to the nutritional pathway. Hunger can lead people to overindulge in high-calorie, high-carbohydrate foods, which can cause insulin resistance. A lack of food or a supply that is inconsistent can exacerbate hypoglycemia. A compensatory mechanism may unintentionally undermine T2DM control through behavioral approaches that are needed to address the instant problem of food insecurity. Medication and supplies for diabetics, for example, would have been redirected to meet dietary needs. When one's basic needs are not being met, it lowers one's sense of self-efficacy and contributes to feelings of depression and diabetes pain. T2DM patients and those at high risk of developing the disease are particularly vulnerable to food insecurity, according to numerous studies. In addition to concerns about overall caloric intake, the nutrient content of the foodstuffs ingested is even more essential than in the prevalent population. Poor metabolic control, severe hypoglycemia among low-income and low-educated populations, lower diabetes self-management behavioral adherence, and poorer glycemic control have been linked to food insecurity in several cross-sectional studies [28].

Diabetes and Food Interventions

T2DM patients who were food insecure were the focus of three studies. To carry out a trial involving blood glucose monitoring, diabetes-appropriate food, self-management ways, and primary care utilization, scientists in California, Texas, and Ohio used a pre- and post-design HbA1c and showed that fruit and vegetable intake, self-efficacy, and adherence to medication all improved as a result of the research. Clinical outcomes did not change as a result of this intervention's results in a randomized controlled trial. In trials, there was a reduction in the BMI but not in HbA1c, as well as better nutritional and psychological effects. T2DM consequences have been examined in relation to supermarket gains and losses. Clinical markers were linked to metrics from a geographic information system based on participants' home locations in a study conducted by Kaiser Permanente Northern California Diabetes Registry. Over the course of four years of tracking store changes in low-income neighborhoods, researchers found that supermarket loss was linked to lower HbA1c trajectories, particularly among those with the highest HbA1c. HbA1c outcomes were slightly better for those with near-normal baseline levels of HbA1c, but only for those with gains in local supermarkets [29].

Using an untamed experiment design, the Pittsburgh Hill/Homewood Research on Eating, Shopping, and Health (PHRESH) examined the consequences of a supermarket and other neighborhood acquisitions on cardiometabolic threat factors in a randomized cohort of citizens from two low-income, predominantly African American neighborhoods [30]. There was greater reported access to nutritious food in the intervention neighborhood (the one that received the supermarket) than in the comparison group. Numerous additional investments have been made in the intervention area since the shop first opened, including greenspace, housing, and commercial space. There will be a public report on the impact of these community investments on blood pressure, BMI, high-density lipoprotein (HDL) cholesterol, and HbA1c shortly. Finally, in the food environment, increased diabetes risk and outcomes are linked to food shortages, inaccessibility, and insecurity when combined, and diabetes-specific nutrition and self-management treatment at food banks or pantries, as well as expanding the presence of grocery stores in low-income neighborhoods, can have a major effect on both the likelihood of developing diabetes and the clinical and psychosocial consequences [30].

Diabetes and medical care

As an SDOH, access, affordability, and therapeutic quality are all factors to consider. Race/ethnicity, socioeconomic position, and location/geographic region in the United States are all significantly connected to these factors.

Diabetes Care and Its Consequences

There is a significant connection between diabetes testing and treatment and the availability of health insurance in population-based research studies. In the United States, uninsured adults are more likely to have undiagnosed diabetes than insured adults. Doctor visits, prescriptions, and emergency department visits were all significantly lower among diabetics who were uninsured than those who were insured. HbA1c values among adolescents and youthful adults with T2DM or T1DM were 0.68% higher when they had state or national health insurance, compared to private insurance, and 1.34% higher when they had no insurance [31].

Although endocrinologists in the United States vary greatly by state and county, many areas with the highest diabetes prevalence and socioeconomic deprivation have gaps in access. Diabetic self-management education programs are more likely to be implemented in areas with a high percentage of high academy graduates, a more elevated ratio of insured people, and a more subordinate unemployment rate. Being insured and having a reliable source of care were found to increase the likelihood that people with diabetes received adequate health care. It is three to five times less likely to get a blood pressure check, HbA1c, or urgent care by uninsured adults who did not have a regular healthcare provider. The HbA1c was the same whether in primary or specialized care for diabetic adolescents and youthful adults who had state or national health insurance and a higher HbA1c was noted among those not having a source provider (primary care or diabetes specialist) [32].

Affordability

Average American healthcare premiums are 2.3 times higher for diabetics compared to nondiabetics. In between 14% and 20% of diabetics, treatment is delayed or postponed because of financial concerns. Diabetes patients receiving insulin may experience rates as high as 25% of the general population. Income, insurance status, and the type of insurance are all linked to cost-related or cost-cutting non-adherence (CRN). Participants in CRN are more likely to be adults with diabetes who are uninsured, and those with diabetes who make $50,000 a year. People with Medicaid or no insurance were three times more viable to develop CRN in a diabetic clinic group compared to those with Medicare. Based on current research, there are several variations in the healthcare system paradigm. Diabetes patients with commercial insurance have a CRN risk that is three times greater than that of diabetic VA patients, and that risk is four to eight times more significant for those with Medicaid, Medicare, or no health insurance at all. CRN has been linked to financial stress, insecurity, and financial constraints. A higher HbA1c indicates a more difficult time controlling diabetes in CRN patients, and they are less able to perform daily tasks. Insulin CRN has been linked to mortality in T1DM patients, including children and adults [33].

Quality

A person's insurance status is the most influential single forecaster of whether or not they are going to fulfill their quality benchmarks for diabetes care. Studies and recommendations from the government show that socioeconomic disparities in healthcare quality persist over time. Among non-Hispanic Blacks and non-Hispanic Whites, Black women had lower odds of acquiring a blended diabetes quality standard than men. There was no progress in diabetes therapy gaps from earlier periods, especially among minorities, women, and younger adults. Disparities in dilated eye examinations, low-density lipoprotein (LDL) testing, HbA1c control, blood pressure management, and statin treatment have been documented in insured contexts. A study of 21 VA hospitals encountered that Blacks with diabetes were better likely than Whites to be treated in lower-performing establishments, which may explain a few of the racial disparities in diabetes quality standards [34].

Diabetes and Healthcare Interventions

Many periodic reviews have found that community health worker (CHW) programs with qualified lay workforces are advantageous for a variety of outcomes among impoverished Hispanics and African Americans with T2DM and associated illnesses. Some regions have paid CHWs as part of the healthcare delivery system. This includes everything from helping patients find their way around the hospital to making sure they show up for appointments, making sure they know what services are available to them when they do, as well as providing them with information about those services. Improved quality of life, fewer emergency visits, hospitalizations decreased costs, and subtle improvements in glycemic control have all been reported as benefits of utilizing home-based or integrated health unit delivery systems for diabetes management. However, a study that used a standard, all-condition CHW intervention and found subtle improvements in diabetes consequences, as well as other health benefits, found that most CHW interventions for adult diabetic populations were diabetes-centered and used structured curricula [35].

Interventions in Organizations

Systematic studies reveal that racial and ethnic minorities have benefited from improved diabetes care thanks to the use of health communication technology (i.e., computerized decision support for providers, patient registries in the electronic health record, reminders, and centralized outreach for diabetes patients overdue for specific services). When programs are designed to help patients overcome obstacles, there is more evidence that self-management treatments are effective. It has been found that diabetes prevention program (DPP) lifestyle change programs recognized by the CDC are adequate in attaining performance standards among Medicaid recipients, and those additional strategies helped to maintain high DPP attendance over 12 months. Studies have shown that a self-management training program for low-income and minority populations is necessary because of illiteracy and the frequent functional limitations caused by diabetes that prevent self-management education from being provided. Clinical outcomes, self-care practices, self-management acquaintance, and problem-solving proficiency in low-income, racial minority, and remote populations are improved by the use of the techniques [3].

Social Environment as a Health Factor

Social capital, cohesion, and support are just a few of the many factors that contribute to a healthy social environment. When it comes to a collective effort, social capital refers to the qualities of social institutions. It is important to note that while mutuality and respect are important in building social capital between people who do not share a common social identity, trust and cooperation are crucial in building social capital within a network that does. A person's health is influenced by macro-level social capital factors such as racism, discrimination, and inclusion or exclusion. In a community, social cohesiveness refers to the degree to which groups are able to work together. Both inequities and social exclusion patterns among certain demographic groups are being addressed, as is the quality of interpersonal relationships and interactions within society as a whole. Social cohesion actions are not just about reducing prejudice and discrimination against economically disadvantaged groups like women and ethnic minorities, but also about fostering social bonds, community ties, and intergroup harmony in the community. In this context, "social support" refers to people's interactions with each other, both formal and informal. Emotional support, physical assistance, informational assistance, and companionship are just a few examples. According to a theory, the positive impacts of social support are buffered by the negative effects of illness or as a means of promoting health [36].

Diabetes Outcomes in a Social Context

Regardless of the quality or portion of social capital, it was found to have a favorable effect on diabetes treatment in a wide range of populations. As a result, it is difficult to derive strong conclusions about the extent of social capital and whether the link is identical for individuals or neighborhoods. Researchers analyzed trust, shared values, willingness to help each other, and social cohesion of neighbors to determine the strength of a community. According to a study, T2DM incidence was found to be 22% lower in neighborhoods with greater social cohesion. Better glycemic control and a higher quality of life are linked to higher levels of social support in diabetes research, whereas a deficiency of social aid has been coupled to higher mortality and diabetes-related intricacies [37].

Studies show that racism and prejudice have an impact on or influence social cohesion, capital, and support. It is a complex system of interconnected elements that strengthen each other, nurturing racial unfairness and encouraging institutional and individual bias in society, all of which have an impact on diabetes incidence. The Multi-Ethnic Study of Atherosclerosis looked for ties between major and day-to-day discriminatory events and diabetes in a mixed sample of 5,310 people ranging in age from middle age to old age. The Black Women's Health Study found a correlation between daily racism and a 31% more heightened risk of diabetes and a 16% more elevated risk of diabetes in women who had experienced the most racism throughout their lifetime. Further research is required to fully comprehend the numerous ways in which the social environment fosters unfairness in diabetes outcomes [38].

Effects of Social Support

No empirical studies have been done to examine the role of social assets or social cohesion interventions on diabetes mellitus consequences, despite a gigantic body of periodicals on the subject. People with T2DM who had more social support had more acceptable glycemic control, better understanding, better treatment compliance, better grade of life, diagnosis understanding and acceptance, and less stress, according to Strom and Egede's comprehensive review of 18 observational studies [39]. T2DM patients who lack social support are at greater risk of death and diabetes-related complications. Improved diabetes-related effects (clinical, self-management, and/or psychosocial behavior modification) were encountered in adults with T2DM in Strom and Egede's review of 16 sociable support intervention studies, and refinements in clinical outcomes (HbA1c, blood pressure, and lipids) appeared independent of the source or delivery. Before the outbreak of the coronavirus disease pandemic, a study was executed to examine the preferences of participants. Telephone-based support and group support were preferable to telephone support for Hispanic diabetics, while African American preferences were more diverse (i.e., telephone, group, and internet). Whites tended to rely more on the media and healthcare professionals, whereas minorities relied more on family and community support [39].

Global Trends in Diabetes Complications

Worldwide, diabetes has become more predominant in the last few decades and now affects 415 million people. As diabetes prevalence rises, so does the number of established and critical diseases in the prevalent population, which could have serious consequences for the quality of life, healthcare demand, and financial costs. End-stage renal disease (ESRD), retinopathy, neuropathy, and lower extremity amputations (LEAs) are among the many burdens associated with diabetes that can be attributed to macrovascular complications such as stroke, coronary heart disease, and peripheral vascular disease, as well as microvascular intricacies like ESRD [40].

Infections, cancer, and liver disease are just a few of the ailments now known to be linked to one another. Diabetes complications may become more important in the future because of a decrease in all-cause and CVD death rates among diabetics. There is a critical lack of quantification of the international burden and divergence in the incidence of diabetes-related comorbidities, despite extensive worldwide evaluations of the growth in diabetes prevalence. Data strategies and population-based analyses to monitor diabetes intricacies are concentrated in North America, Europe, and other high-income countries; low- and middle-income countries, where the incidence of diabetes is rapidly increasing, have little to no access. To compare trends around the world, there is a need for standardized diagnosis and tracking of diabetes-related complications [41].

Complications of the Macrovascular System

The leading cause of death and disability among diabetics is CVD. It is only a matter of time before there are more cases of CVD as a result of diabetes. In diabetics, the risk of CVD appears to have decreased since the 1990s, according to some studies. Diabetes patients still hold a two- to four-fold more elevated hospitalization risk for major CVD events and CVD-related procedures than people without diabetes, despite these advancements in diabetes care. In most high-income countries, mortality rates from CVD have decreased in the general population. Even in patients with diabetes, CVD is still the foremost cause of mortality, with diabetics having a two- to four-fold higher CVD mortality rate than non-diabetics [42].

Several studies have shown a decrease in CVD-related deaths among diabetics. CVD mortality in the United States decreased by 53% between 1988 and 1994, and there was a decrease in the risk gap between diabetics and non-diabetics from 2010 to 2015. Between 2000 and 2011, Australia's CVD mortality rates dropped by 50%, while Iceland's CVD mortality rates dropped by 46% between 1993 and 2004. Despite a 44.1% and a 17.1% decrease in in-hospital mortality for acute myocardial infarction (AMI) and stroke in Canada between 1992 and 1999, diabetics were still 1.6 times more probable than non-diabetics to die from these possibilities. Patients with T1DM in Switzerland and Australia have also seen similar declines in CVD mortality [7].

Complications of the Microcirculation

Sudden complications: Because of their high costs and high morbidity and mortality rates, acute diabetes complications such as DKA, hyperglycemic hyperosmolality (HHS), lactic acidosis, and hypoglycemia can be prevented to a large extent. Researchers found that 29% of people in aged care were living with diabetes in the United States. The reasons for the rise in acute complications in the United States remain a mystery at this point.

Lower extremity amputation: LEAs are a major concern because of the medical, financial, and emotional consequences. For this reason, and others, the prevalence of LEAs is an essential indicator of the significance of various preventative measures, such as blood glucose management, CVD threat management, screening, and therapy for those at increased risk of foot problems. Between 1982 and 2011, the prevalence of LEAs decreased across a wide range of demographics, as shown by population-based studies. One study in Spain found significant reductions in blood glucose levels, while the other in Australia found no such declines. Because of decreases in major LEAs in all 13 nations and significant territories of countries with data, there appears to be a decrease in overall LEA incidence. Many countries have even reported increases in minor LEAs, which have shown smaller relative declines. Consequently, it is expected that the number of small LEAs done to prevent major LEAs in the clinical setting will go up. There are still large differences in LEA rates within communities. LEA rates in the United States, for example, have dropped significantly among the elderly, with only minor decreases among the young and middle-aged. The number of LEAs continues to be higher among non-Whites and male residents in the United States, with significant geographic differences [43].

Neuropathy: Since periodic population surveys do not collect data on changes in neuropathy prevalence or incidence, we have no idea how the disease is changing over time. Between 2000 and 2014, the US Diabetes Surveillance System (USDSS) found that the rate of neuropathy-related hospitalizations increased by 42.1%. Changes in neuropathy coding and increased awareness of neuropathy among diabetics are likely to have an impact on these data. The prevalence of distal symmetrical polyneuropathy in T1DM patients diagnosed between 1970 and 1974 was lower than that in those diagnosed between 1965 and 1969, according to the Pittsburgh Epidemiology of Diabetes Complications Study.

The most common long-term complications of diabetes are diabetic neuropathies. This wide range of disorders affects a variety of parts of the nervous system and has a wide variety of clinical symptoms [10]. Diabetic neuropathy must be diagnosed and treated as soon as possible for a variety of reasons, including those indicated in Table 2.

**Table 2:** Reasons for the requirement of early diagnosis and treatment of diabetic neuropathy

Reasons
1. Diabetic neuropathy is an excluding the diagnosis. Patients with diabetes may have non-diabetic neuropathies, which may be treated with particular treatments.
2. Diabetic neuropathy with symptoms has a variety of therapy choices.
3. Diabetic peripheral neuropathies might be asymptomatic in up to 50% of cases. Patients are at increased risk for injury to their insensate foot if they are not diagnosed and preventative foot care is not provided.
4. Autonomic neuropathy may be diagnosed and treated, which can help to alleviate symptoms, prevent complications, and enhance the quality of life [10].

Distal symmetric polyneuropathy (DSPN) and diabetic autonomic neuropathies, notably cardiovascular autonomic neuropathy (CAN), are by far the most researched types of diabetic neuropathy. Diabetic neuropathy comes in a variety of types, including atypical diabetic neuropathy. Neuropathies comparable to diabetic neuropathies may occur in people with prediabetes. Because there are no medications that target the underlying nerve damage, diabetes avoidance is essential. Diabetic neuropathy screening is extremely important in clinical practice since it may identify the earliest stages of neuropathy and allow for early management. Although screening for uncommon atypical types of diabetic neuropathy may be necessary, the most prevalent kinds found in practice are DSPN and autonomic neuropathy. These kinds have the strongest available evidence in terms of therapy.

Diffuse somatic neuropathy of the distal symmetric sensorimotor kind is the most prevalent neuropathy that affects diabetics. Pain, paresthesia, hyperesthesia, dysesthesia, proprioceptive deficiency, loss of feeling, and muscular weakening and atrophy are common symptoms of a mixed sensorimotor dysfunction. Autonomic nerve function is often compromised, and in certain cases, a single nerve fiber is primarily impacted. Small nerve fiber damage causes painful neuropathy whereas big myelinated fiber function is preserved. Proprioception, vibration sensing threshold, and deep tendon reflexes are all retained. Motor and proprioceptive deficits result from neuropathy that mostly affects big nerve fibers [44].

Autonomic dysfunction, test type and number, age-related normative values, presence or absence of diabetic autonomic neuropathy (DAN) signs and symptoms, and patient cohorts studied all have a significant impact on the prevalence of DAN.

Patients with T1DM and T2DM were both found to have prevalence rates of CAN that ranged from 1% to 90%, while Dimitropoulos found prevalence rates of CAN that ranged from 20% to 70%. In community-based population research, however, 16.7% of people had autonomic neuropathy, which was characterized by one or more aberrant heart rate (HR) variability test findings.

Ziegler et al. found that 25.3% and 34.3% of T1DM and T2DM patients, respectively, had CAN in a multicenter study published in 1992 (more than two of six abnormal autonomic function tests). At least three of six autonomic function tests were abnormal in at least 16.8% of T1DM patients and 22.1% of T2DM patients, with O'Brien et al. finding a comparable prevalence rate in T1DM patients. The prevalence of the condition was similar for both groups.

While Solders et al. found low sensory nerve conduction and autonomic dysfunction in about 25% of 144 diabetic children, Karavanaki found evidence of reduced papillary adaptation in darkness in 13.8% of children with diabetes and 5.8% of controls, with 50% of these showing the elicited.

In the Diabetes Control and Complications Trial (DCCT), 1.65% of patients with diabetes for more than five years exhibited abnormal HR variability at baseline. The prevalence climbed to 6.2% in individuals with diabetes for more than five but less than nine years, and to 12.2% in those with diabetes for more than nine years.

For patients with T1DM and T2DM, 16.6-20% of patients have confirmed CAN (defined as abnormalities of at least two cardiovascular HR results). This prevalence may increase to 65% with increasing aging and diabetes duration in unselected clinical studies. Patients with T1DM and 44% of those with T2DM who are 40-70 years old, as well as 35% of those with T1DM and 65% of those with T2DM who have had the disease for a long time, may have prevalence rates as high as 38% and 44%, respectively.

About 7% of people with T1DM and T2DM are diagnosed with CAN at the time of diagnosis. The annual increase in the prevalence of CAN in T2DM and T1DM has been estimated to be about 6% and 2%, respectively.

DAN may produce gastrointestinal abnormalities in all parts of the gastrointestinal tract, including delayed esophageal transit (50%), gastroparesis (40%), disordered small and large intestine motility with diarrhea (20%), and constipation (20%). With erectile dysfunction (35-90%) and retrograde ejaculation, the incidence of organic sexual dysfunction is equally significant (32%) [45].

Patients with T1DM (43-85%) and those with T2DM (25%) are more likely to experience bladder problems. According to Tang's systematic review of 19 studies on children and adolescents with T1DM, the prevalence of DAN ranged from 16% to 75% for cardiovascular nerve function tests and 8% to 16% for pupillometry investigations in the juvenile population. Glycemic control, the duration of diabetes, and abnormalities in autonomic testing in young people have all been linked, according to some studies. It appears that retinopathy and nephropathy are linked to DAN data from the adult population, the only studies that have looked at the link between DAN and other microvascular problems in juvenile diabetics [45].

End-stage renal disease: Approximately 80% of all cases of ESRD worldwide are attributed to diabetes or hypertension. Russian, Philippine, Malaysian, Republic of Korean, Mexican Jalisco, Singaporean, and Australian cases of diabetes-related ESRD have increased significantly between 2002 and 2015, as have cases in Bosnia and Herzegovina, Scotland, and the Philippines. At an average annual rate of 11%, the United States grew over the same period. During the same time period, the incidence of diabetes-related ESRD decreased in Austria, Belgium, Finland, Denmark, and Sweden. According to the data, the preponderance of T1DM and T2DM in various countries is likely to be a contributing factor. Diabetic populations are not included in these figures. According to a national analysis of Chinese participants, the incidence of ESRD among those with T2DM decreased by about 6% per year between 2000 and 2012. When it comes to diabetes, the incidence of ESRD in the United States decreased by 28% between 1990 and 2010. As a result of more inclusive standards for inciting renal replacement therapy early on and a significant reduction in cardiovascular complication rates that improved diabetic morbidity and mortality, this reduction was more diminutive than for other diabetes-related complications such as stroke, LEAs, and demise from hypoglycemia. Dialysis initiation is more common among diabetics of certain races and ethnicities than among those of other races and ethnicities. ESRD treatment rates fell by 28%, 22%, 14%, and 13% among American Indian/Alaska Natives, Hispanic, non-Hispanic Whites, and non-Hispanic Blacks with diabetes in the United States from 2000 to 2013 [46].

Asian diabetics' risk of developing ESRD remained stable over the same time period. According to the United States Renal Data System (USRDS), T2DM was to blame for 91% of all new cases of ESRD. While it is true that T1DM is less common than T2DM, there are diagnostic ambiguities that can lead to people with diabetes or those on insulin being incorrectly diagnosed as having T1DM, making changes in the recurrence of characterized ESRD in T1DM less obvious. Despite this, a study on ESRD in eight European countries or regions, as well as non-indigenous Canadians and Australians, found a decrease in the incidence of T1DM-related ESRD between 1998 and 2002. Cohort studies have shown that children diagnosed in the past few decades have a lower risk of ESRD than those diagnosed in the 1960s and 1970s, even though there were no nationwide sampling methods for T1DM populations as there are for T2DM. T1DM-related ESRD may be on the decline due to the increased use of renin-angiotensin system inhibitors and statin therapy in this cohort at younger ages, as well as recent advances in insulin delivery methods. ESRD caused by T1DM has more than doubled in Taiwan between 1999 and 2010 [47].

Retinopathy: In diabetics, diabetic retinopathy impacts one-third of patients and is the foremost cause of blindness. Despite the prevalence of diabetic retinopathy, data on incidence trends in the general population are lacking. Only a few studies have been able to establish a consistent annual incidence of retinopathy over time. According to population-based studies, the incidence of diabetic retinopathy has decreased by 50-67% since the 1990s. A meta-analysis of 28 studies including 27,120 T1DM and T2DM diabetics found that the pooled incidence of proliferative diabetic retinopathy was inferior in 1986-2008 than in 1975-1985. In the Pittsburgh Epidemiology of Diabetes Complications Study, the incidence of proliferative diabetic retinopathy decreased from 38% in 1965-1969 to 26.5% in 1975-1980 [48]. The incidence of various complications of diabetes varied globally, as can be seen in Table 3.

**Table 3:** Trends in diabetes mellitus complications

Micro and macrovascular complications	Country	Period	Trends
Cardiovascular disease [7]	USA	1988-1994	Incidence decreased by 53%
Cardiovascular disease [7]	Australia	2000-2011	Incidence decreased by 50%
Neuropathy [10]	USA	2000-2014	The rate of hospitalization increased by 42.1%
End-stage renal disease [46]	USA	1990-2010	Incidence decreased by 28%
Retinopathy [48]	Globally	1990	Drop-in incidence by 50-67%
Lower extremity amputation [43]	Spain and Australia	1982-2011	Decrease in incidence

Mortality

All-Cause Mortality

Diabetes-related mortality rates are often approximated using vital statistics systems, the effectiveness of which is influenced by coding methods, diabetes prevalence, and country-level diabetes awareness. As a result, to accurately monitor death rates in diabetic populations, rates should preferably be computed among specified cohorts of people who have been diagnosed with diabetes. However, statistics on diabetes-related cause-specific mortality and all-cause mortality are complex to evaluate since they originate from a limited number of nations in Europe, North America, Asia, and Australia. Because the methodology used in the research varies, these statistics should be understood rather than a comparison of absolute rates, since patterns through time across nations varies. Nonetheless, since the late 1980s, there has been a steady fall in diabetes-related mortality. Ranging between 2000 and 2009, Taiwanese women with diabetes saw a 4% relative drop in mortality, compared to a 37% relative decline among Canadian women with diabetes between 1996 and 2009 [49].

Studies comparing diabetic and non-diabetic populations demonstrate that the relative disparities amongst the two groups are narrowing with time, but diabetes-related increased risk persists, even in more recent time periods. The mortality rate ratio in Ontario, Canada fell from 1.90 in 1996 to 1.51 in 2009, and comparable drops have been seen in the United Kingdom, the United States, and Australia. When compared to those without diabetes, people with T1DM have a 3-18-fold increased risk of mortality. Several studies, however, have observed ongoing improvements in mortality rates. For example, between 1950 and 2009, the number of fatalities related to T1DM in the United States decreased significantly across all age categories. Between 1968 and 1969 and 2008 and 2009, diabetes-related mortality before the age of 20 years decreased by 61%, according to a study by the CDC. When comparing death rates between individuals diagnosed with T1DM as a kid between 1965 and 1969 and those diagnosed between 1975 and 1979, the difference is significant. Outside of the United States, Japan and Finland recorded decreases of 69% and 8%, respectively. The lower decrease in Finland is most likely due to the country's lower absolute mortality when compared to Japan. In Norway, fatality rates for those diagnosed with T1DM before the age of 15 years fell by 81% (from 286 to 53 per 100,000 person-years) between 1973 and 1982, compared to those diagnosed between 1999 and 2012. In Australia, fatality rates among those diagnosed with T1DM before the age of 45 fell by 33% between 2000 and 2011 [50].

Mortality (Non-cardiovascular)

Diabetes is linked to a number of other non-cardiovascular causes of mortality. Diabetes was linked to an increased risk of death from renal disease, liver disease, cancers, infections, falls, digestive system disorders, mental health issues, pneumonia, external causes, intentional self-harm, and nervous system disorders, according to a meta-analysis study. Trends in non-cardiovascular mortality have only been seen in a few studies. Between 1988 and 1994, the rate of cancer-related fatalities in the United States fell by 16% per 10 years, whereas non-vascular rate-oriented non-cancer deaths decline by a lesser percentage (8% every 10 years). Between 2000 and 2011, age-standardized mortality rates (ASMRs) for CVD, all-cause, and diabetes in Australia declined dramatically, whereas cancer-related ASMRs among adults with T1DM and T2DM remained stable. Cancer has overtaken heart disease as the leading cause of mortality among diabetics, according to data from the same national registry in Australia, rising from 25% to 35% of all deaths between 1997 and 2010. In the United States and Taiwan, similar results have been observed. This is critical in view of the rising incidence of diabetes, which is occurring at the same time as the population ages, and the latter is a risk factor for diabetes as well as cancer [51].

Emerging Complications

The total years of life lived with diabetes have grown due to an augmentation in diabetes prevalence during the 1980s, paired with reduced mortality among diabetics. Longer life expectancy among diabetics has prompted the discovery of additional problems, such as infections, cancer, and cognitive impairment. When compared to persons without diabetes, people with diabetes have a superior risk of urinary tract infections, tuberculosis, severe infections (gram-positive), and tropical illnesses. It is unclear if the incidence of infections in diabetic populations has altered over time. According to the National Vital Statistics System data, the percentage of deaths in people with diabetes decreased from 3.1% in 1999 to 2.7% in 2010, while the percentage of deaths in people without diabetes increased from 4.5% to 4.9%; in both categories, respiratory tract infections account for the largest proportion of mortality. According to the National Nursing Home Survey data, the age-standardized proportion of people residing in a nursing home having nosocomial infections increased from 6.1% to 10.3% in people with diabetes between 1999 and 2004, while it increased from 6.0% to 8.5% in people without diabetes. Between 2008 and 2012, there was a 61.3% rise in sepsis hospitalization rates in Spain, and with revisions in the International Classification of Diseases, Ninth Revision (ICD-9) clinical modification codes, it is tough to follow changes in sepsis over time [52].

Diabetes increases the risk of anxiety, major depressive disorder, eating disorders, dementia, schizophrenia, and a variety of disability cases like reduced instrumental or basic activities, working disabilities, mobility loss, etc., according to a growing body of evidence. For the reason that there is a lack of prospective data with appropriate follow-up for many of these disorders, it is impossible to say if the risk has changed over time. Two studies have looked at depression patterns throughout time. Between 2001 and 2011, the occurrence of depression amongst T2DM patients (hospitalized) in Spain grew dramatically, from 3.5% to 5.8%, with increases being much larger in women. Antidepressant usage was more prevalent in persons with diabetes in Finland than in those without, and the use of these medicines grew more quickly in people with diabetes between 1997 and 2007, especially among younger people with T2DM. In terms of physical disability, statistics from the United States reveal that the frequency of both impaired instrumental activities of daily living and mobility has remained constant over the last several centuries, whereas disability of work has decreased in 1997 from 23.8% to 17.9% in 2006, before rising in 2011 to 19.7%. In terms of relative trends in disability rates, the non-diabetic group had comparable patterns, although the absolute rates over time were lower [53].

## Conclusions

Regardless of the fact that now the review does include SDOH intervention studies for accommodation, the constructed and nourishment ecosystem, and healthcare, there appears to be little research on the impact of interventions on diabetes aimed at income, education, toxic environmental exposures, occupation, social capital, and social cohesion around the world. In contrast, a clinical setting is insufficient to account for SDOH effects across the body. The core causes of SDOH must be addressed via structural and regulatory reforms. New research is also required that focuses on therapies that tackle the fundamental causes of diabetes inequities rather than compensatory therapy that helps people live with injustices. We also raised the issue of a lack of data on the global situation of diabetes exacerbations incidence, especially in low- and middle-income countries. This data gap is mostly owing to the lack of population-based techniques for monitoring healthcare spending since cohort studies and surveys are often insufficient for evaluating diabetic difficulties. It is also difficult to assess trends in complications because of the many reporting methodologies, definitions of complications, and strategies to identify people with diabetes. Implementing standardized reporting approaches and implementing realistic processes might help to enhance future monitoring of diabetes complications trends over the globe.
